# Study on the electrical-thermal properties of lithium-ion battery materials in the NCM622/graphite system

**DOI:** 10.3389/fchem.2024.1403696

**Published:** 2024-04-12

**Authors:** Hao Li, Xv Wu, Sheng Fang, Mei Liu, Shansong Bi, Ting Zhao, Xiangjun Zhang

**Affiliations:** ^1^ National Power Battery Innovation Center, China GRINM Group Corporation Limited, Beijing, China; ^2^ China Automotive Battery Research Institute Co., Ltd., Beijing, China; ^3^ General Research Institute for Nonferrous Metals, Beijing, China

**Keywords:** lithium-ion battery, thermal runaway, differential scanning calorimetry, state of charge, electrolyte

## Abstract

The phenomenon of fire or even explosion caused by thermal runaway of lithium-ion power batteries poses a serious threat to the safety of electric vehicles. An in-depth study of the core-material thermal runaway reaction mechanism and reaction chain is a prerequisite for proposing a mechanism to prevent battery thermal runaway and enhance battery safety. In this study, based on a 24 Ah commercial Li(Ni_0.6_Co_0.2_Mn_0.2_)O_2_/graphite soft pack battery, the heat production characteristics of different state of charge (SOC) cathode and anode materials, the separator, the electrolyte, and their combinations of the battery were investigated using differential scanning calorimetry. The results show that the reaction between the negative electrode and the electrolyte is the main mode of heat accumulation in the early stage of thermal runaway, and when the heat accumulation causes the temperature to reach a certain critical value, the violent reaction between the positive electrode and the electrolyte is triggered. The extent and timing of the heat production behaviour of the battery host material is closely related to the SOC, and with limited electrolyte content, there is a competitive relationship between the positive and negative electrodes and the electrolyte reaction, leading to different SOC batteries exhibiting different heat production characteristics. In addition, the above findings are correlated with the battery failure mechanisms through heating experiments of the battery monomer. The study of the electro-thermal properties of the main materials in this paper provides a strategy for achieving early warning and suppression of thermal runaway in batteries.

## Introduction

In recent years, the need to address climate change and energy security has driven the large-scale application of lithium-ion batteries in the energy sector, and lithium-ion battery technology has become the core support technology for electric vehicles, renewable energy access and smart grids. Energy density is the core indicator of lithium-ion battery, high energy density can enhance product competitiveness, while reducing the cost per watt-hour. Thanks to the optimisation of material system and battery structure design, the energy density of commercial lithium-ion batteries has gradually increased to the current 360 Wh/kg. However, along with the significant increase in energy density, the safety of lithium-ion batteries is becoming more and more prominent. The instability of the internal materials of high energy density batteries makes it possible for external abuse conditions or internal defects to cause side reactions in the materials and continue to generate heat to promote the generation of more side reactions (Huang et al., 2021). The by-products contain a large number of flammable gases, and the continuous reaction causes the battery temperature to rise, and when the temperature reaches a critical value, it eventually triggers thermal runaway of the battery, resulting in a fire or even an explosion (Feng et al., 2018). The battery thermal runaway is the most important factor in improving the safety of batteries. Improving battery safety requires the establishment of a battery thermal runaway prevention, warning and prediction mechanism, which relies on the system design from the *system - cell - material* three levels, the battery thermal runaway reaction mechanism is the basis of the design. Revealing the thermal runaway mechanism requires an in-depth study of the thermal stability of materials and the reaction chain during the thermal runaway process.

Nickel-cobalt-manganese (NiCoMn) ternary materials are commonly used as cathode materials for high energy density lithium-ion batteries. As the proportion of Ni element increases, the thermal stability of anode materials decreases, the onset temperature of exothermic reaction decreases, and the amount of exothermic heat increases (Noh et al., 2013; Bak et al., 2014; Gong, Wang, and Sun, 2017). Bak(Bak et al., 2013) studied a variety of ternary cathode materials using *in situ* X-ray diffraction (XRD) and mass spectrometry, and indicated that the migration of nickel and cobalt under heating leads to structural changes in the materials and the release of oxygen. Mei et al. (Zhang et al., 2023) found that during the thermal runaway process of lithium iron phosphate batteries, the heat production of the anode material is not significant due to the strong stability of the P-O bond. The commonly used negative electrode material is graphite, and the electrolyte will form a solid electrolyte interface (SEI) on the surface of graphite, and the main components of the SEI are based on Li_2_CO_3_, LiF, ROCO_2_Li, ROCOO_2_Li, and PEO-Li et al. (Hess, 2017). Dahn (Dahn, 1991) studied the thermal behavior of graphite anode, and the results showed that the SEI started to decompose around 100–130°C. After the membrane structure was destroyed, the electrolyte came into direct contact with the embedded lithium anode, releasing a large amount of heat (Andersson and Edström, 2001; Andersson et al., 2002). The decomposition of SEI is accompanied by the production of gases such as ethylene, oxygen and carbon dioxide (Gachot et al., 2012). Some researchers have also studied the reaction between lithium-embedded graphite and binder (Park et al., 2014). The electrolyte has been called the blood of the battery. Most common commercial electrolytes use carbonate as the solvent and LiPF_6_ as the lithium salt. The covalent bond bonding between cyclic carbonates is higher than that between chain carbonate chains, leading to the former’s high thermal stability (Jiang and Dahn, 2004). The onset temperature of thermal decomposition of the electrolyte varies with the type of solvent and the composition of the additives, and the oxygen and water content will significantly reduce the thermal stability of the electrolyte (Sheng et al., 2024).

Compared to when each material is present individually, chemical reactions occur between interacting materials within the confined system of the battery. These reactions lead to a further decrease in the thermal stability of the system. After the addition of electrolyte, the electrolyte-ternary material hybrid system, the thermal stability decreases compared to the ternary material without electrolyte. Feng et al., 2014 (Y. Wang et al., 2022) studied based on NCM systems decomposed the battery thermal runaway process into six major exothermic reactions, and proposed the existence of material crosstalk between the positive and negative electrodes and the electrolyte. A study by Volkswagen AG found that interactions between different battery components are the main source of heat during thermal runaway and gave an energy release diagram (Lenz et al., 2023).

In addition, the state of charge significantly affects the thermal stability of the battery. SOC is not only related to the onset temperature at which the battery undergoes thermal runaway but is also closely linked to the intensity of thermal runaway. The researchers have separately investigated the thermal stability of Li(Ni_1/3_Co_1/3_Mn_1/3_)O_2_ (Zhang et al., 2024), LFP and NCA(Golubkov et al., 2015), respectively, and found that the thermal runaway trigger temperature decreases with increasing SOC, and the maximum temperature increases with increasing SOC, with the trigger temperature decreasing more significantly for batteries that are overcharged to more than 100% SOC. He (He et al., 2023) et al. pointed out that the heat production of mono and binary systems other than the positive electrode is positively correlated with the state of charge. Li et al., 2019. studied Li (Ni_0.8_Co_0.1_Mn_0.1_)O_2_/SiC batteries and found that batteries with higher than 50% SOC are more prone to thermal runaway, but also pointed out that the self-heating onset temperature and thermal runaway trigger temperature of batteries with different SOCs do not change much.

It can be seen that revealing the thermal runaway reaction process requires not only the study of the thermal stability of the materials and the effect of thermal interaction between the materials, but also an in-depth study of the changes in the thermal runaway reaction chain under different SOC states. Thus, the relationship between battery safety and material electro-thermal properties can be established. In this paper, the thermal stability of the positive and negative materials, electrolyte and separator, and their interactions under different states of charge were investigated using the differential scanning calorimetry (DSC) test with NCM622/graphite flexible pack batteries as the research object. It reveals the interactions and reaction sequences between the positive and negative electrode materials and electrolyte in different lithium-embedded states, as well as the influence of the negative side reaction on the positive side reaction in the case of limited electrolyte content, which provides a direction for improvement in the early warning and prevention of thermal runaway of the battery.

## Materials and methods

The NCM622/graphite soft pack lithium-ion batteries used in the experiment were manufactured by China Automotive Battery Research Institute Co., Ltd., with a design capacity of 24 Ah. The basic parameters of the batteries are shown in Table 1. The batteries were calibrated for capacity on a charge/discharge meter, and the SOC of these batteries was regulated according to the GB 38031-2020 standard, and the SOC states included: 0%, 50%, 75%, and 100%.

**TABLE 1:** Main parameters of the batteries used in the experiment.

Item	Value
Anode	Li(Ni_0.6_Co_0.2_Mn_0.2_)O_2_
Cathode	Graphite
Electrolyte composition	EC:EMC = 3:7, LiPF_6_ 1 mol/L
Rated voltage range	2.8–4.2 V
Rated capacity	24 Ah
Exterior dimensions	308*115*6.5 mm

**FIGURE 1:**
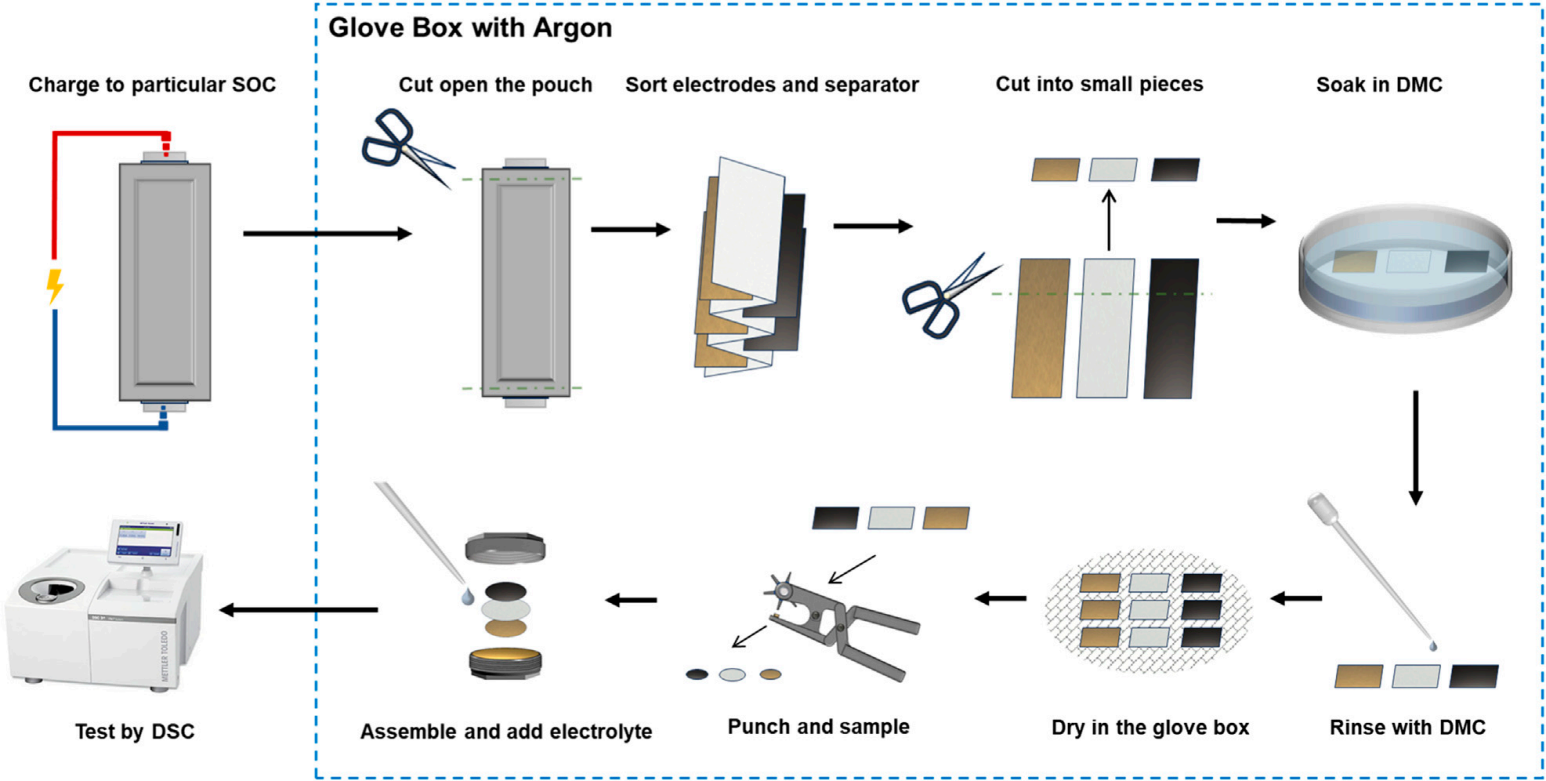
Preparation and DSC test of main material samples of soft pack battery.

The battery samples were disassembled in an argon glove box to separate the main materials, and the sample processing flow is shown in Figure 1. The outer aluminium-plastic film of the battery was cut using ceramic scissors, and the positive and negative electrodes and the separator were separated using ceramic tweezers. The separated positive and negative electrodes and separator were cleaned separately, soaked in dimethyl carbonate (DMC) for 40 min to remove the residual lithium salts and other substances on the surface, rinsed again and dried in the glove box, and sealed for storage.

Tests were carried out using a METTLER TOLEDO DSC 3 device with a high-pressure crucible made of gold-plated steel. Electrode samples for DSC tests are often prepared by scraping, but some scholars have suggested that this affects the macrostructural properties of the material and ignores the effect of foils in thermal runaway, suggesting that the test be carried out directly on the electrode sheet (Inoue and Kazuhiko, 2017a; Inoue and Kazuhiko, 2017b; Zhang et al., 2023). In this study, a pair of punching forceps were used to place the electrode samples on the foil. In this study, the electrode piece and the separator were processed into round pieces for DSC test using punching forceps. The electrodes were placed flat into the bottom of the crucible to ensure good contact, where the diameter of the positive and negative electrodes was 3.5 mm and the diameter of the separator was 4 mm. Corresponding electrolytes were added dropwise to keep the ratio of active substances in the positive electrode, negative electrode, and electrolyte the same as that in the battery samples. The above operations were carried out in an argon glove box. The temperature range of the DSC experiments was 25°C–350°C, with a temperature increase rate of 5°C/min and a nitrogen atmosphere.

Additionally, the thermal runaway mechanism of pouch battery cells was investigated. A thermal radiation heating chamber was utilized to continuously heat the battery cells at 100% SOC up to 200°C with subsequent insulation. Thermocouples were installed to monitor the temperature changes.

## Results and Discussion

### Self-generated heat study of a single material

In this study, we start from a single component of a soft pack lithium-ion battery and extend it to a binary combination, ultimately modelling the case of negative electrode-separator-positive electrode + electrolyte in a soft pack battery in a crucible. The heat absorption and exothermic peaks were named and numbered based on the experimental group, using the order of the temperature at which the peaks start.

**FIGURE 2:**
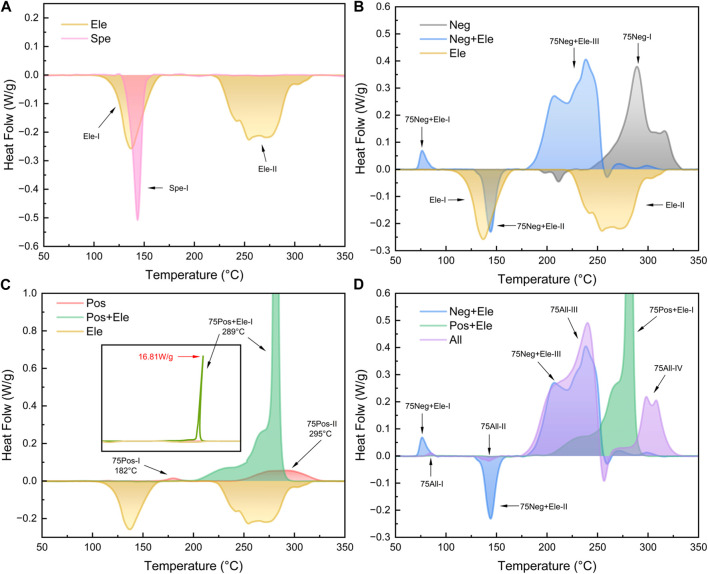
DSC test results of 75% SOC cell **(A)** Separator and electrolyte; **(B)** Negative electrode and electrolyte; **(C)** Positive electrode and electrolyte; **(D)** All component groups.

The thermal characteristic curves of the electrolyte and the separator are demonstrated in Figure 2A. The DSC curve of the electrolyte exhibits two heat absorption peaks, Ele-I at 131°C and Ele-II at 276°C, corresponding to the gasification of the solvent. The boiling points of both solvents were increased due to the use of sealed crucibles (the theoretical boiling points of ethylene carbonate and ethyl methyl carbonate are 243°C and 108°C (Eshetu et al., 2013)). The separator shows a heat absorption peak Spe-I at 138°C–160°C, which represents the contraction and melting of the separator (Liu et al., 2020). The temperature range of the Ele-I peak encompasses the Spe-I completely, and their positions are highly coincident. In fact, in the later experiments, it can be observed that the two are always superimposed and difficult to distinguish from each other.

For the study of positive and negative electrode materials, we start with the 75% SOC battery material. As shown in Figure 2B, for the graphite negative electrode piece alone, there is a major exothermic peak at higher temperature (289°C) 75Neg-I. In the test NCM622 positive electrode showed strong stability (Ren et al., 2018), and only two smaller exothermic peaks were produced at 75% SOC (Figure 2C). This suggests that a single component positive or negative electrode sheet will not have a large thermal effect, and even if there is a large heat production such as 75Neg-I, the trigger temperature is still high. The reaction that occurs when the positive and negative materials are heated individually is not the one that causes the intense heat production in the cell.

### Self-generated thermal characteristics of multi-material combinations

When the electrolyte was introduced into the electrode system with 75% SOC, the heat production peak pattern of the binary system changed significantly, as shown in Figures 2B,C. The exothermic quantities of Group 75Neg + Ele and 75Pos + Ele had a significant increase, especially in the Group 75Pos + Ele. After the addition of electrolyte, the positive electrode shows a strong exotherm, forming the 75Pos + Ele-I peak with a maximum heat flow of 18 W/g in a very narrow interval of 281°C–297°C. This involves the oxygen-releasing behavior of the NCM positive electrode, where the released oxygen reacts strongly with the electrolyte and emits heat (Bak et al., 2014; He et al., 2024). When comparing Group 75Neg + Ele with 75Neg, after adding electrolyte, the exothermic peak at 76°C is related to the decomposition reaction of SEI (Hess, 2017). After the SEI decomposition and rupture, the leaked lithiated graphite directly contacted with the electrolyte, and the reaction between the electrolyte and lithium-embedded graphite and bonding agent will occur (Forestier et al., 2016) The reaction is similar to the formation of SEI. The reaction is similar to the SEI generation process, but at this temperature SEI cannot be stabilized, the reaction will continue until the reactants are consumed. Group 75Neg + Ele main exothermic peaks are wide, distributed in the temperature range of 175°C–250°C. The main exothermic peaks of Group 75Neg + Ele’s are broader, distributed in the temperature range of 175°C–250°C. At this point the position of the main peak, 75Neg + Ele-III, advanced to 246°C and underwent a change in shape. To the left of the main peak a weaker shoulder peak 75Neg + Ele-II with a peak position of 205°C appears.

There is a clear difference in the reactions that occur between the positive and negative electrodes and the electrolyte (P. Huang et al., 2020). The reaction between the negative electrode and the electrolyte has a low onset temperature and a wide range of exothermic temperatures. On the contrary, the main reaction occurring between the positive electrode and the electrolyte occurs near 290°C, with a relatively high onset temperature and a narrow range of reaction temperatures, presenting a sharp peak. These features have important reference value for us to explore the self-heating reaction process of the battery.

In order to investigate the self-generated heat of the full cell under the heating condition, the positive electrode-separator-negative electrode were stacked in the crucible from top to bottom and the electrolyte was added to infiltrate the whole system, and the curves were obtained as in Figure 2D. The curves of Group 75All showed five main characteristic peaks. Among them, the peak 75All-I/II/III onset temperatures and temperature ranges show similar results to those of the corresponding peaks in Group 75Neg + Ele, which indicates that the onset temperature of the reaction between the negative electrode and the electrolyte is still lower than that of the positive electrode in the full-cell combination. No strong exothermic peaks corresponding to 75Pos + Ele-I were found between 280°C and 295°C, and wider double peaks appeared at higher temperatures (298/307°C). The disappearance of the strong exothermic peaks corresponding to Pos + Ele-I may be related to the exhaustion of the electrolyte. This indicates the temporal nature of the reaction, where the positive reaction process will be affected by the negative reaction process.

### Characteristics of self-generated heat in positive and negative electrode materials with different electrolyte contents

**FIGURE 3:**
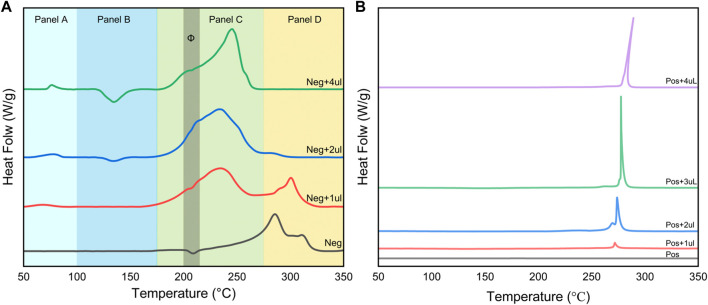
Effect of electrolyte addition on DSC signals of negative **(A)** and positive **(B)** electrode slices.

The experimental results in the previous section show that the consumption of electrolyte by the negative electrode side reaction will affect the onset temperature and exothermic strength of the reaction between the positive electrode and the electrolyte under the condition of certain electrolyte content. In this section we focus on examining the effect of electrolyte on the positive and negative electrode materials, and the experiment is still based on the main material of the battery with 75% SOC. In Figure 3, we observe the heat generation behavior of the positive and negative electrodes with different volumes of the same electrolyte added.

Figure 3A divides the reaction between the negative electrode and the electrolyte into four temperature intervals, which correspond to different physicochemical processes. Panel A demonstrates that the exothermic peak corresponding to the decomposition of the SEI becomes more pronounced and the amount of exothermic heat increases as the electrolyte addition increases. This indicates that the increase in electrolyte content will increase the amount of SEI produced. The gasification reaction of solvent EMC occurs in the temperature interval of Panel B. Panels C and D are the temperature intervals where the exothermic reaction of the negative electrode dominates. Without adding electrolyte after cleaning, the exothermic reaction mainly exists above 270°C, where the exothermic reaction of lithiated graphite and binder occurs (Forestier et al., 2016). After the introduction of electrolyte, the exothermic peak near 240°C then appeared and became the more dominant exothermic reaction; the peak near 300°C gradually weakened to disappearing with the increase of electrolyte content. This means that the reactions occurring in Panel C and Panel D are competitive, as shown by the fact that the embedded lithium negative electrode will be the first to react with the electrolyte at lower temperatures, forming an exotherm in Panel C. When the amount of electrolyte is low, the remaining negative electrode active material will continue to react with the binder in the temperature interval corresponding to Panel D. When enough electrolyte is added to completely consume the embedded lithium negative electrode, the peak of the reaction ceases to appear. In addition, in the Φ zone in Panel C, the slopes of several curves show significant changes, which implies that two reactions with different exothermic rates do exist here.

Figure 3B shows that the exothermic peak of the reaction between the positive electrode material and the electrolyte changes less with the increase of electrolyte content. The onset temperature of the reaction peak increases slightly with the content, rising from 250°C without electrolyte to 278°C. This is due to the increase of the solvent, which enhances the heat absorption of the gasification. At the same time, the exothermic amount undergoes a significant enhancement with the increase of electrolyte content. The tip of the peak for Pos+4uL is shifted to the high-temperature position, representing that the reaction at this point is intense and fast. The reaction between the positive electrode and the electrolyte differs only in the amount of heat discharged, which is controlled by the amount of electrolyte.

Electrolyte content plays a key role in the spontaneous exothermic reaction of lithium-ion batteries. For the negative electrode, a gradual transition of the main exothermic reaction (peak position) of the system occurs as the amount of electrolyte added increases. For the positive electrode, it is mainly the enhancement of the exothermic intensity caused by increasing the amount of electrolyte. This will guide our investigation of the spontaneous heat production characteristics of different SOC battery host materials, because to a certain extent, different SOCs correspond to different proportions of active substances in the host materials.

### Characteristics of self-generated heat from battery materials with different SOC

**FIGURE 4:**
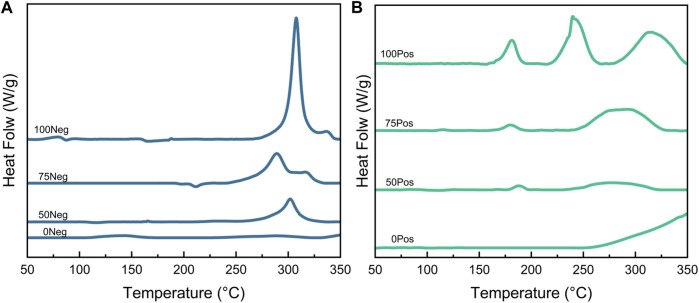
Negative **(A)** and positive **(B)** DSC test curves for different SOCs.

**FIGURE 5:**
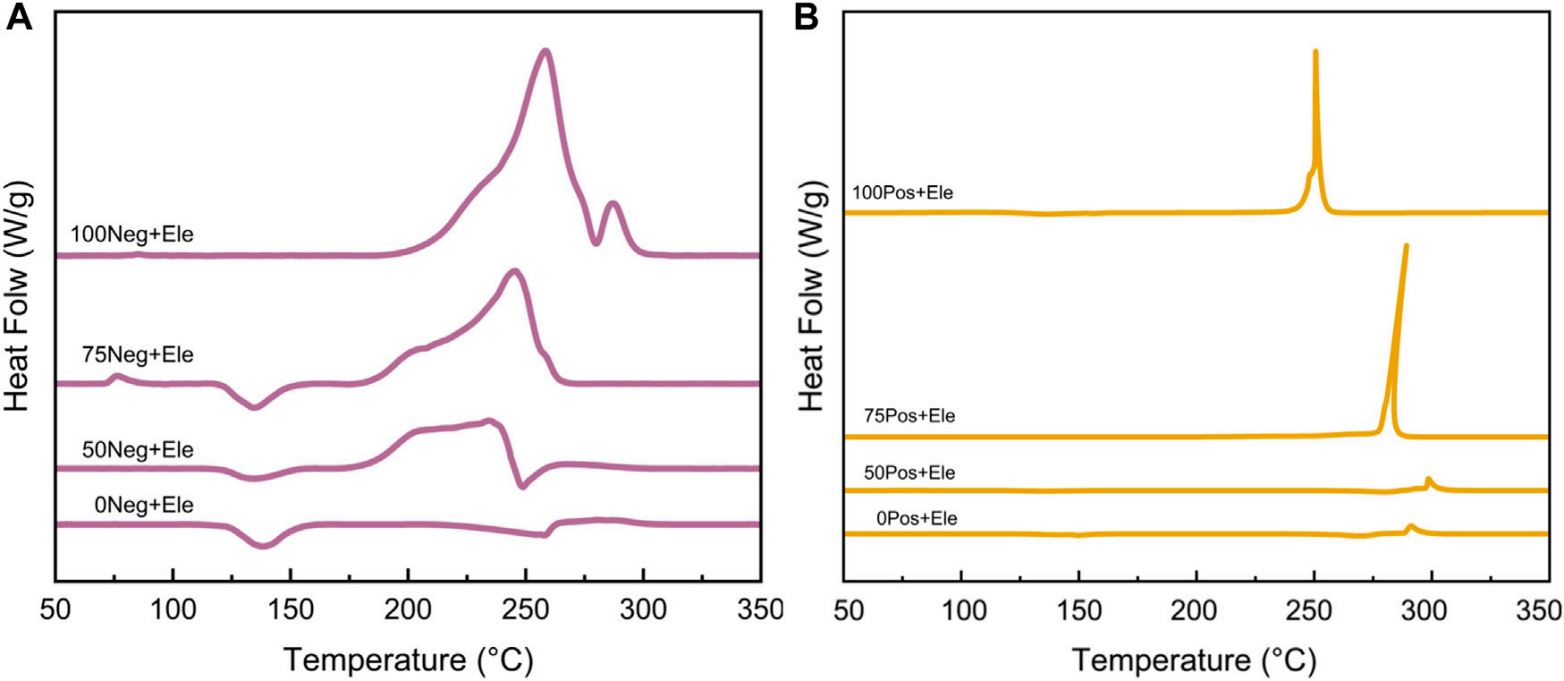
DSC test curves of different SOC negative **(A)** and positive **(B)** electrodes plus electrolyte groups.

**FIGURE 6:**
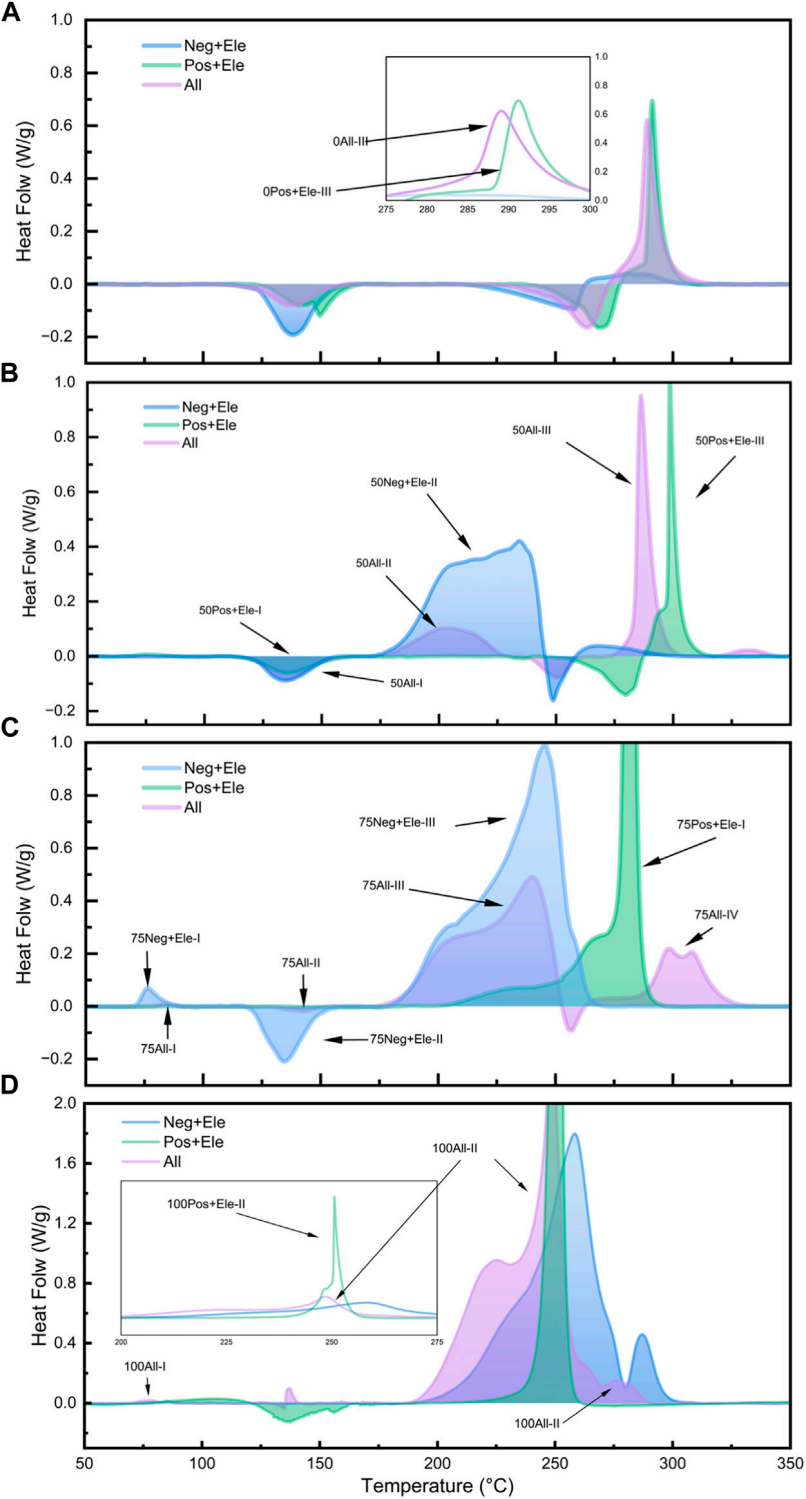
DSC test curves for different SOC all component groups: 0% SOC **(A)**; 50% SOC **(B)**; 75% SOC **(C)**; 100% SOC **(D)**.

In this study, four SOC states were set to investigate the relationship between the state of charge and the spontaneous exothermic characteristics of lithium-ion batteries, the four states of charge are: 0% SOC, 50% SOC, 75% SOC, and 100% SOC, and the experimental results are shown in Figure 4, Figure 5 and Figure 6, respectively.

Figure 4A demonstrates the exotherm of the negative electrode at different SOCs. With the increase of SOC, the embedded lithium in the graphite negative electrode deepens. As a result, the stability decreases, leading to a gradual increase in heat production. This process produces a cluster of obvious exothermic peaks at higher temperatures (–300°C). Similarly, the increase of SOC corresponds to the deepening of the degree of delithiation of the negative electrode, which leads to the instability of the crystal of the negative electrode material, and it is more likely to change the crystal shape and exotherm under the heating condition (G. Zhang et al., 2020). The DSC curve of the negative electrode is relatively more stable than that of the positive electrode. The DSC curve of the negative electrode is relatively more complicated, with 2–3 exothermic peaks in a wide temperature range and changes with SOC.

The thermal characteristics of the positive and negative electrode materials with different SOCs after the addition of electrolyte are demonstrated in Figure 5. After the addition of electrolyte in the experimental system, the negative electrode samples then underwent a significant exotherm between 175°C and 250°C, and the amount of exotherm increased with the increase of SOC. In the above temperature range, the intensity of the first peak with the increase of SOC always stays around 0.4 W/g, which is presumed to be related to the substances in the negative electrode that do not change with SOC, such as the binder, *etc.*,; while the second peak undergoes a more obvious enhancement from almost 0–1.8 W/g, which corresponds to the reaction between graphite with different degrees of lithium embedded and electrolyte. The reaction between the positive electrode and the electrolyte with different SOCs all mainly exists a sharp exothermic peak; with the increase of SOC, this peak gradually increases and slightly shifts to the low temperature direction, as shown in Figure 5B. In summary, the thermal stability of both positive and negative electrode materials has a tendency to decrease with the increase of SOC, and the reaction with electrolyte is more intense.

Figure 6 illustrates the heat production in the All system with different SOCs and compares the relationship between Group Neg + Ele, Group Pos + Ele, and Group All at different SOCs. In the 0% SOC and 50% SOC groups, the heat release of the All group is mainly concentrated in the high temperature region of 275°C–300°C, and the peak exothermic curve has a similar curve characteristic to that of the Group Pos + Ele with the same SOC, which accounts for more than 50% of the overall heat release. Combined with the previous discussion, the exothermic peak here corresponds to the exothermic peak of the reaction between the positive electrode sheet and the electrolyte in Group All.

However, when the SOC reaches 75%, the curve of the Group All changes significantly in the high-temperature region, and the spike in the high-temperature region is replaced by a weaker double spike; the exothermic amount of the positive electrode with the electrolyte no longer occupies a dominant position, and the main exothermic process occurs in the temperature interval of 175°C–250°C. In this SOC state, the positive electrode undergoes a reaction with the electrolyte, and the exothermic reaction of the electrolyte involves interaction with the positive electrode. In this SOC state, the continuous reaction between the lithium-embedded negative electrode and the electrolyte drastically reduces the residual amount of electrolyte reacting with the positive electrode material, which in turn leads to a significant decrease in the proportion of heat production from the reaction between the positive electrode and the electrolyte. All the above results describe the competitive relationship between the positive and negative electrodes to the electrolyte in the experimental system. The active substances in the positive and negative electrodes have their own exothermic reaction intervals, which is their intrinsic property, and the difference in the charge state leads to the difference in the relative content of the active substances, which endows the positive and negative electrodes with the corresponding competitive ability. Among them, the positive electrode active substance is more sensitive to the reaction and produces obvious thermal effect at lower SOC, but the reaction trigger temperature is higher; while the negative electrode active substance occupies the advantage of low reaction temperature, and takes the lead in seizing the electrolyte to react during the warming process at a certain SOC. These properties lead to the substance distribution relationship in the experimental system.

In addition, in the DSC test of the Group 100All, the peak of the reaction between the positive electrode and the electrolyte at high SOC is shifted to a low temperature of 258°C. This peak is ahead of the exothermic peak of the reaction between the negative electrode and the electrolyte at 260°C, which is an advantage over the competition, and therefore results in the exothermic peak of the Group 100All appearing at 251°C, which is the result of the competition.

### Trigger heat production characteristics of cell

**FIGURE 7:**
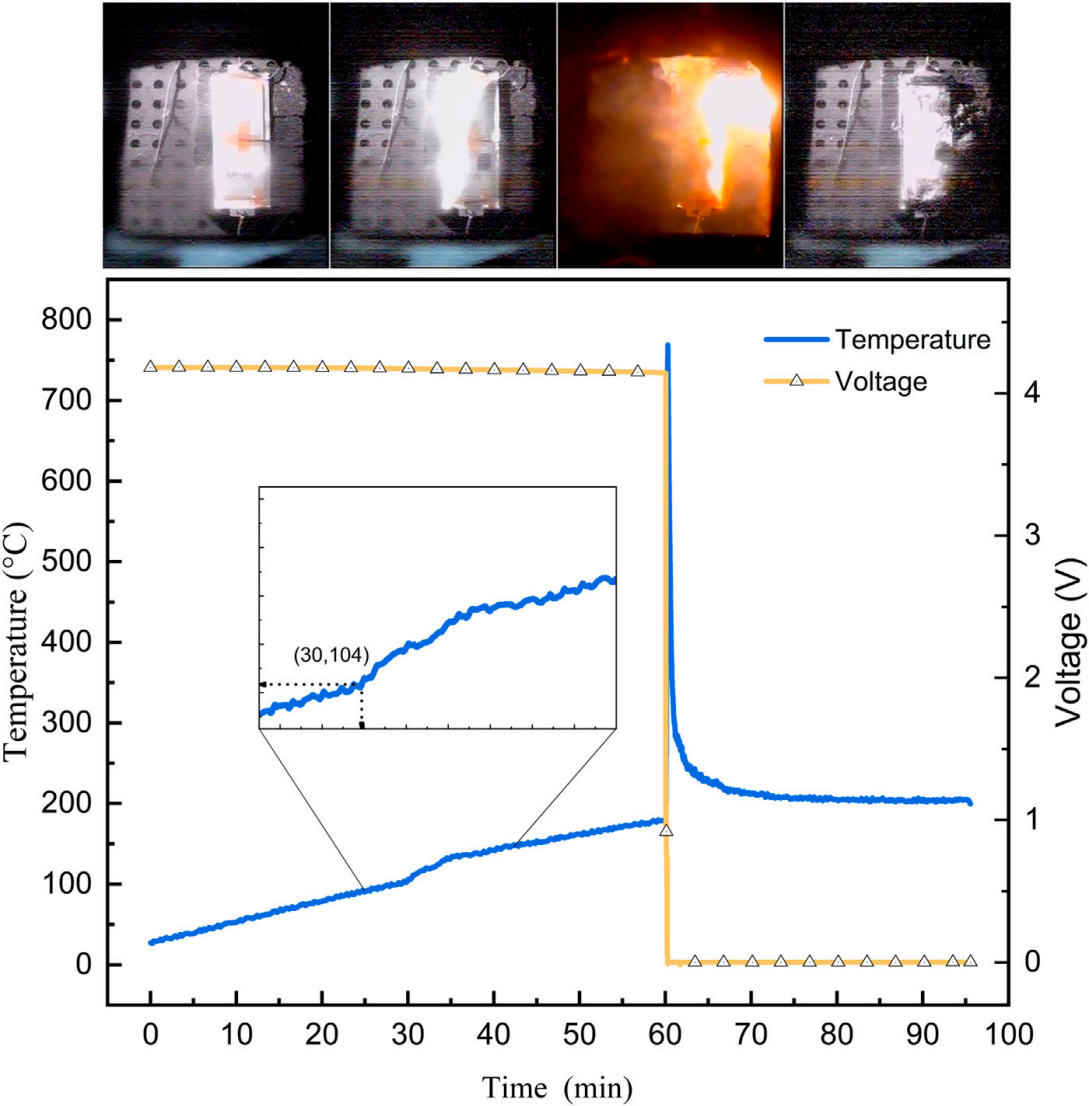
Experimental phenomena and temperature-voltage curve of cell heating test.

The temperature and voltage changes during the heating-triggered battery thermal runaway experiment are shown in Figure 7. After 56 min of heating in the test chamber, the temperature reached 200°C and was maintained for insulation. At 60 min of insulation, the battery underwent severe thermal runaway, with the aluminum-plastic film inflating, rupturing, igniting, and the voltage dropping sharply to 0 V.

Through the recorded data from the deployed thermocouples, we can roughly observe the internal changes within the battery. After maintaining a constant temperature for 30 min, self-heating occurred successively in various parts of the experimental battery, causing deviations in the temperature rise trend from the original environmental conditions. After a period of heat accumulation, this ultimately led to thermal runaway, with the triggering temperature falling within the range of 165°C–200°C. This aligns with experimental conclusions at the material scale: prior to thermal runaway, spontaneous reactions within the battery sustain a prolonged period of slow heat generation, primarily corresponding to reactions between the negative electrode active material and the electrolyte. Under conditions of ample electrolyte, when the temperature exceeds 200°C and reactions between the positive electrode and the electrolyte occur, rapid and intense exothermic reactions will ensue.

Based on the previous experiments, we have found that the exothermic reaction between the negative electrode and the electrolyte occurs over a wide temperature range, with significant heat generation but relatively slow kinetics. Leveraging this characteristic, early detection of heat accumulation through monitoring methods is essential for issuing alarms, which first requires the Battery Management System (BMS) to accurately monitor battery temperature and identify abnormal temperature rises. Additionally, within the battery pack, a Battery Thermal Management System (BTMS) is installed, which can enhance heat dissipation through reasonable temperature control strategies such as air cooling or liquid cooling to prevent thermal runaway (Jin et al., 2014; Xu et al., 2017). However, the rapid exothermic reaction between the positive electrode and the electrolyte is difficult to interrupt promptly. Therefore, efforts should be made to avoid the battery reaching this stage of self-heating (Afzal et al., 2023), or timely warnings should be issued for evacuation and withdrawal to minimize losses (Shen et al., 2024). Due to the reaction between the positive electrode and the electrolyte shifting towards lower temperatures at high SOC, intense thermal runaway can occur at lower temperatures. This poses requirements for the BMS in terms of battery consistency judgment and charge-discharge strategies, requiring the adoption of more sensitive and effective capacity monitoring strategies while avoiding overcharging and over-discharging of the battery (Cheng et al., 2011). Furthermore, we have observed that the presence of the electrolyte significantly reduces the thermal stability of both the positive and negative electrodes, lowering the temperature of heat generation and increasing the amount of heat released. Therefore, from the perspective of battery design, developing safer and more stable electrolytes (Han et al., 2023) or using solid-state electrolytes (J. Wang et al., 2023) are also important measures to prevent accidents.In summary, the exothermic reaction associated with the negative electrode exhibits significant heat generation over a long period, while the reaction associated with the positive electrode is rapid and intense. These two different characteristics provide research directions and solutions for restraining thermal runaway and its propagation.

## Conclusion

In this work, based on the DSC test technique, the heat production characteristics of different embedded lithium batteries’ positive and negative materials, diaphragm and electrolyte are investigated by disassembling different SOC batteries, revealing the electro-thermal characteristics of the materials and the reaction time sequence during the thermal runaway process. The study shows that during the thermal runaway process of the battery, the decomposition of the SEI film firstly occurs at around 80°C, followed by the gasification of the electrolyte components. From 175°C, the reaction between the negative electrode and the electrolyte occurs, which is the main heat accumulation mode in the early stage of the battery thermal runaway. When the temperature reached a certain critical value, the reaction between the positive electrode and the electrolyte was triggered, resulting in a strong thermal runaway. By studying the effect of electrolyte addition on the heat production of positive and negative electrode materials, the competitive relationship between the reaction of positive and negative electrode materials and electrolyte in batteries was analyzed. The low reaction trigger temperature of the negative electrode will consume the electrolyte at a lower temperature, which inhibits the positive electrode from reacting with the electrolyte and thus affects the thermal runaway heat production process. This competitive relationship and the timing of the reactions lead to differences in the exothermic characteristics of thermal runaway in different SOC batteries. Based on this, early warning of abnormal temperature rise in the early stage of the battery and avoidance of high SOC can be adopted to prevent the occurrence of thermal runaway in Li-ion batteries.

## Data Availability

The raw data supporting the conclusion of this article will be made available by the authors, without undue reservation.
